# Effects of the antimicrobial peptide WK3 on diarrhea, growth performance and intestinal health of weaned piglets challenged with enterotoxigenic *Escherichia coli* K88

**DOI:** 10.29219/fnr.v65.3448

**Published:** 2021-05-11

**Authors:** Licong Zhang, Tao Guo, Na Zhan, Taotao Sun, Anshan Shan

**Affiliations:** Institute of Animal Nutrition, Northeast Agricultural University, Xiangfang District, Harbin, People’s Republic of China

**Keywords:** WK3, weaned piglets, diarrhea, growth performance, intestinal

## Abstract

**Background:**

Antibiotics are very effective for treating diarrhea in weaned pigs, but the global prohibition of antibiotics makes it urgent to find an alternative to antibiotics.

**Objective:**

An experiment was conducted to determine the antimicrobial activity of a linear trpzip-like β-hairpin antimicrobial peptide WK3 *in vivo* and to assess its effects on growth performance and intestinal health.

**Design:**

Thirty-two piglets were weaned at 21 days and housed in individual metabolic cages, which were randomly divided into four groups and were maintained on a corn-soybean meal-based basal diet. Group 1 included a blank group. Groups 2, 3, and 4 were orally infected by feeding with Enterotoxigenic *Escherichia coli* (ETEC) K88, which was followed by saline treatment (group 2), enrofloxacin injection at a dose of 2.5 mg/kg (group 3), and WK3 injection at a dose of 2 mg/kg (group 4). The experiment lasted for 6 days, and feed and water were provided ad libitum.

**Results:**

Both WK3 and enrofloxacin effectively attenuated diarrhea and improved growth performance of piglets. Compared with the control group, WK3 significantly improved the villus height in the ileum (*P* < 0.05) but did not affect the villus height in the duodenum or jejunum. Additionally, we did not observe any obvious difference in crypt depth or villus height/crypt depth among the duodenum, jejunum and ileum (*P* > 0.05). WK3 also reduced the numbers of *Enterococcus* spp (*P* < 0.01) in the cecal contents, and the number of *Enterobacterium* spp tended to decrease (0.05 < *P* < 0.1). Moreover, the jejunal mucosa of the WK3 group exhibited lower interleukin-1α (*IL-1a*; *P* < 0.01), toll-like receptors-4 (*TLR-4*; *P* < 0.05), and myeloid differentiation primary response 88 (*MyD88*; *P* < 0.01) messenger ribonucleic acid (mRNA) expression levels. The jejunum of the WK3 group also exhibited an increased antioxidant capacity, reduced concentration of malondialdehyde (MDA; *P* < 0.05), and enhanced superoxide dismutase (SOD) activity (*P* < 0.05).

**Conclusions:**

WK3 has the potential to replace antibiotics as a new generation feed additive.

## Popular scientific summary

- The antimicrobial peptides were regarded as potential substitutes for antibiotics.
- WK3 showed ideal antimicrobial activity, protease stability *in vitro*.
- To explore the antimicrobial activities of the antimicrobial peptide WK3 *in vivo*, the diarrhea model of 24 piglets created by ETEC K88 has been established.
- WK3 could reduce the diarrhea rate and improve the growth performance by improving the immune function and intestinal microflora and intestinal morphology, reducing the expression of inflammatory cytokines.

Piglets experience various types of stressors that decrease their resistance to pathogens and make them susceptible to various diseases after weaning. Diarrhea is reported as a challenging health problem in weaned piglets, which has brought about huge economic losses in modern swine production (1, 2). *Escherichia coli* (*E. coli*), which can cause diarrhea, is a predominant species of facultative anaerobe found in the gut of pigs (3). Enterotoxigenic *E. coli* (ETEC) K88 is one of the most common pathogens causing diarrhea in postweaning piglets. After colonizing the small intestine, ETEC produces enterotoxins that stimulate intestinal inflammation, thereby causing diarrhea (4). Piglets can no longer obtain passive immunity from sows after weaning, which raises their susceptibility to ETEC infections.

Antibiotics have been widely used to enhance the growth of piglets, as well as to reduce their susceptibility to diseases (5). However, the overuse and abuse of antibiotics have resulted in the emergence of bacterial resistance and drug residues in animal-derived products, thereby endangering human health (6). As a result, there is a need to develop antibiotic substitutes.

Antimicrobial peptides (AMPs), also known as host defense peptides, are important components of biological innate immunity, which have displayed broad-spectrum antimicrobial activity against bacteria, fungi, viruses, parasites, and even cancer cells (7, 8). The antimicrobial activities of AMPs are closely related to their amino acid composition and physical–chemical properties, such as net positive charge, flexibility, amphipathicity, and hydrophobicity (8–10). The mechanism of action of AMPs against pathogens mainly involves membrane permeabilization, which completely differs from the mechanism of antibiotics (11); thus, bacterial resistance is highly unlikely to appear. AMPs have been reported to have potential to enhance the growth performance and immune functions of piglets (12). A series of linear trpzip-like β-hairpin AMPs were synthesized by our laboratory by positioning paired Trp residues at nonhydrogen-bonded sites and Lys residues at hydrogen-bonded sites according to the sequence template (WK)*n*^D^ PG(KW)*n*-NH_2_ (*n* = 1, 2, 3, 4, 5). Among the series of AMPs, (WK)_*n*_^D^ PG(KW)_n_-NH_2_ with *n* = 3 has the most lethal effect on Gram-positive and Gram-negative bacteria. WK3, which is composed of 14 amino acids, has good antimicrobial activity, protease stability, and shows low hemolytic activity and cytotoxicity *in vitro* (13).

In this study, we used ETEC K88 to create a pig model of inflammation to explore the antimicrobial activity of WK3 *in vivo* and to assess its effects on growth performance and intestinal health, thereby providing a theoretical basis for the replacement of antibiotics with AMPs.

## Materials and methods

### Materials

WK3 (WKWKWK^D^PGKWKWKW-NH_2_, 2.07 kDa) was synthesized using the solid-phase method with Fmoc chemistry by Synpeptide Co, Ltd (Shanghai, China), with a purity of ≥95%. The peptide was then stored at −80°C until further analysis.

ETEC K88 was obtained from the China Institute of Veterinary Drug Control (Beijing, China). After being shaken in 10 mL Luria-Bertani (LB) broth at 37°C for 24 h, ETEC K88 was screened on a LB agar plate. A single colony was inoculated into 50 mL LB broth, was incubated on a rotary shaker overnight at 37°C, and then, the culture was diluted on LB agar for bacterial enumeration.

### ETEC challenge in weaned piglets

Thirty-two piglets (Duroc × Landrace × Yorkshire) were purchased from a local commercial pig farm (Harbin, China). All piglets were weaned at 21 days and housed in individual metabolic cages in a temperature-controlled nursery room (23–25°C). Twenty-four piglets were orally administered with 10^8^ CFU/mL of ETEC K88 (100 mL) for 3 days after 4 days of prefeeding. The piglets were then monitored for symptoms of diarrhea. Diarrhea developing in all 24 piglets was used as the main criterion for successfully establishing the diarrhea model.

### Experimental design and diets

Thirty-two piglets were randomly divided into four groups (groups 1, 2, 3, and 4) with eight piglets in each group. All piglets were maintained on a corn-soybean meal-based basal diet without any antibiotics. The experimental diets were formulated to meet the requirements of National Research Council nutrition (NRC2012) (14). The diet ingredients and the nutritional levels of the diets are displayed in Table 1. Group 1 did not receive any treatment, serving as a blank. Groups 2, 3, and 4 were orally infected with ETEC K88, which was followed by saline treatment for group 2 (control), enrofloxacin injection at a dose of 2.5 mg/kg body weight for group 3 (Enro), and WK3 injection at a dose of 2 mg/kg body weight (WK3) for group 4. The dosage for WK3 was set based on results of several earlier experiments involving mice. The experiment was carried out for 6 days, and feed and water were provided *ad libitum*.

**Table 1:** Composition of experimental diets for piglets (as feed basic)

Ingredient (g/kg)	Content
Corn	695.6
Soybean meal, dehulled	176.5
Fish meal	30.0
Soybean oil	15.0
Wheat bran	50.0
Dicalcium phosphate	8.0
Limestone	7.8
Salt	3.5
L-Lysine-HCl, 98%	2.6
Vitamin and mineral premix^1^	10.0
Choline chloride	1.0
**Nutritional content (g/kg)**	
Digestion energy (DE) (MJ/kg)	13.9
Metabolizable energy (ME) (MJ/kg)	12.9
Crude protein (CP)	166.5
Ca	6.5
Total P	5.6
Lys	10.6
Met	2.8
Met + Cys	5.5

1 Provided the following per kilogram of diet: 8,000 IU vitamin A, 2,000 IU vitamin D_3_, 30 IU vitamin E, 1.5 mg vitamin K_3_, 1.6 mg vitamin B_1_, 1.5 mg vitamin B_6_, 12 μg vitamin B_12_, 15 mg D-pantothenic acid, 20 mg nicotinic acid, 80 mg Zn (ZnSO_4_), 100 mg Fe (FeSO_4_), 20 mg Cu (CuSO_4_), 25 mg Mn (MnSO_4_), 0.3 mg I (KI), and 0.2 mg Se (Na_2_SeO_3_).

All nutritional content were calculated values.

### Sample collection

The weights of piglets were recorded at the start and end of the experiment. Feed intake was recorded daily throughout the entire experimental period. The piglets were anesthetized with an intravenous injection of sodium pentobarbital (50 mg/kg body weight) and bled by exsanguination. The animal procedures were approved by the Ethical and Animal Welfare Committee of Heilongjiang Province, China. Cecal contents were collected and then immediately placed in liquid nitrogen before being preserved at −80°C for microbial analysis. The middle sections of the duodenum, jejunum, and ileum tissues were isolated, and then flushed with 0.9% physiological saline; the tissues were then fixed with 10% formaldehyde-phosphate buffer and kept at 4°C for use in the microscopic assessment of mucosal morphology. The other portion of the mucosal scrapings from the jejunum was sealed into pockets and preserved at −20°C until being used for the evaluation of antioxidant status. The mucosal layer of the jejunum was scraped off and immediately immersed in liquid nitrogen prior to storage at −80°C until ribonucleic acid (RNA) was extracted.

### Analytical methods

The diarrheal index was based on the fecal consistency scoring system (0, normal; 1, soft feces; 2, mild diarrhea; and 3, severe diarrhea), as described previously. A fecal score of 2 or 3 was considered clinical diarrhea (15).

Diarrhea index = sum of diarrhea scores for each group of piglets during the trial period/(number of days tested × number of piglets per group);

Diarrhea incidence (%) = number of piglets with diarrhea per treatment during the trial period/(number of days tested × number of piglets per group) × 100%;

Average daily gain (ADG) = (weight at the end of the experiment – weight at the beginning of the experiment)/ number of experimental days;

Average daily feed intake (ADFI) = (total weight of the feed – weight of remaining feed)/number of experimental days;

Feed weight gain ratio (F/G) = average daily feed intake/average daily gain.

Formalin-fixed intestinal samples were prepared using paraffin embedding techniques. After hematoxylin-eosin staining, the samples were imaged by film under 100´ magnification using the American Moticam 3000 photomicrography imaging system. The intestinal villus height and crypt depth were measured using the Motic Images Advanced 3.2 Pathological Image Analysis System. Intact villi were selected and measured in five copies for each slice, and mean values were calculated.

Total deoxyribonucleic acid (DNA) was extracted from the cecal samples using the TIANamp Stool DNA kit (Tiangen Biotech Ltd, Beijing, China) by following the manufacturer’s instructions. The quality of DNA was evaluated by nano Photometer (Implen GmbH). The genus-specific 16S ribosomal RNA-targeted primer sequences are shown in Table 2. For identification of bacterial groups, real-time polymerase chain reaction (RT-PCR) was performed using the SYBR^®^ Premix Ex TaqTM II system (TaKaRa^®^ Bio Catalog). Dissociation analyses of the PCR products were conducted to confirm the specificity of the resulting PCR products. Quantification was performed in duplicate, and the mean values were calculated. The results were reported as log10 16S ribosomal DNA gene copies per gram fresh matter.

**Table 2:** Primers used for quantitative real-time polymerase chain reaction to detect bacterial numbers

Targeted bacterial group	Amplicon size (bp)	Primer sequence (5’–3’)^1^	Annealing temperature (°C)
Total eubacteria	200	F:CGG(C/T)CCAGACTCCTACGGG R:TTACCGCGGCTGCTGGCAC	58
*Lactobacillus* spp.	341	F:AGCAGTAGGGAATCTTCCA R:CACCGCTACACATGGAG	62
*Enterobacterium* spp.	.195	F:CATTGACGTTACCCGCAGAAGAAGC R:CTCTACGAGACTCAAGCTTGC	60
*Bifidobacteria* spp.	243	F:TCGCGTC(C/T)GGTGTGAAAG R:CCACATCCAGC(A/G)TCCAC	58
*Enterococcus* spp.	144	F:CCCTTATTGTTAGTTGCCATCATT R:ACTCGTTGTACTTCCCATTGT	60

1 F = forward; R = reverse.

Total RNA was extracted from approximately 100 mg of frozen jejunal mucosa using the total RNA kit (E.Z.N.A.^®^, Omega Biotek, Inc.) following the manufacturer’s instructions. The RNA concentration was measured using a spectrophotometer, and the purity was ascertained by the A260: A280 ratio. The total RNA from each sample was reversed transcribed into DNA (cDNA) using the Prime Script^®^ RT reagent kit (TaKaRa^®^ Bio Catalog), according to the manufacturer’s instructions, and the resulting complementary DNA (cDNA) was used for RT-PCR. SYBR Green I RT-PCR kit (TaKaRa^®^ Bio Catalog) was used to measure the messenger RNA (mRNA) expression of cytokines (interleukin-1α [*IL-1α*], interleukin-1β [*IL-1β*], interleukin-8 [*IL-8*], toll-like receptors 4 [*TLR-4*], and myeloid differentiation primary response 88 [*MyD88*]) relative to the expression of the β-actin endogenous control. Specific primers were designed using the Primer Express^®^ software (PE Applied Biosystems) and were synthesized by Sangon Biological Engineering Co. Ltd. The primer sequence data are presented in Table 3. For analyses on an ABI PRISM 7500S thermal cycler (Applied Biosystems), the reactions were performed with 1 μL of first-strand cDNA and 0.2 μM of sense and antisense primers in a final volume of 10 μL. The samples were briefly centrifuged and run on a PCR thermocycler using the default fast program (one cycle at 95°C for 30 sec, followed by 40 cycles at 95°C for 5 sec and 60°C for 34 sec). Relative gene expression levels were determined using the 2^-ΔΔCt^ method.

**Table 3:** Sequences of the oligonucleotide primers used for quantitative real-time polymerase chain reaction

Genes	GenBank number	Primer sequence (5’–3’)^1^	Product length (bp)
Interleukin-1α (*IL-1α*)	NM_214029	F:CTGAAGAAGAGACGGTTGAG R:GCACTGGTGGTTGATGAC	162
Interleukin-1β (*IL-1*β)	NM_214055	F:GGCCGCCAAGATATAACTGA R:GGACCTCTGGGTATGGCTTTC	70
Interleukin-8 (*IL-8*)	NM_213867	F:CTGGCTGTTGCCTTCTTG R:GTCCACTCTCAATCACTCTC	163
*β-Actin*	AY550069	F:ATGCTTCTAGGCGGACTGT R:CCATCCAACCG ACTGCT	211
Toll-like receptors 4 (*TLR-4*)	NM_001293316.1	F:CAGATAAGCGAGGCCGTCATT R:TTGCAGCCCACAAAAAGCA	113
Myeloid differentiation primary response 88 (*MyD88*)	NM_001099923.1	F:TGGTAGTGGTTGTCTCTGATGCAA R:TGGAGAGAGGCTGAGTGCAA	80

1 F = forward; R = reverse.

The concentration of malondialdehyde (MDA), the activity of superoxide dismutase (SOD) and glutathione peroxidase (GSH-PX), and the total antioxidant capacity (T-AOC) of the jejunum were measured using commercial kits (Bioengineering Company of Nanjing Jiancheng, Nanjing, China).

### Statistical analysis

The indices were analyzed by analysis of variance (ANOVA) and Duncan’s multiple range tests using SPSS 19.0 statistical software. The data were expressed as mean ± standard error of the mean (mean ± SEM). The level of significance was accepted at *P* < 0.05, and 0.05 < *P* < 0.1 was considered as a trend.

## Results

### Diarrhea and growth performance

The effects of WK3 on diarrhea and growth performance are shown in Table 4. We observed that WK3 and Enro significantly reduced the diarrhea index (*P* < 0.01) and diarrhea rates (*P* < 0.01) of piglets. The ADG (*P* < 0.05) and ADFI (*P* < 0.01) of piglets in the WK3 and Enro groups were significantly higher than those in the control group but were not significantly different from the blank group. Injection with WK3 did not significantly affect the feed weight gain ratio (F/G) relative to the control group.

**Table 4:** Effects of antimicrobial peptide WK3 on diarrhea and growth performance of weaned piglets

Item	Treatment				Standard error of the mean (SEM)	*P*-value
	Blank	Control	Enro	WK3		
No. of piglets	8	8	8	8		
Diarrhea index^1^	0.53^a^	2.01^c^	1.53^b^	1.52^b^	0.14	<0.001
Diarrhea rate	3.47^a^	80.56^c^	46.53^b^	50.00^b^	7.43	<0.001
Average daily weight gain (ADG), kg	0.50^b^	0.34^a^	0.57^b^	0.53^b^	0.03	0.003
Average daily feed intake (ADFI), kg	1.06^b^	0.83^a^	1.15^b^	1.07^b^	0.03	0.001
Feed conversion ratio (F/G)	2.23	2.80	2.04	2.05	0.18	0.430

1 Diarrhea index = sum of diarrhea scores for each group of piglets during the trial period/(number of days tested × number of piglets per group).

a,b,c Means in the same row with different superscripts differ (*P* < 0.05).

All the values are expressed as means ± SEM.

### Villus height and crypt depth

The effects of WK3 on villus height and crypt depth of the intestines are shown in Table 5. We found that WK3 significantly improved the villus height of the ileum (*P* < 0.05), but did not affect the villus height of the duodenum or jejunum. Moreover, crypt depth and villus height/crypt depth did not show obvious differences among duodenum, jejunum and ileum (*P* > 0.05). In addition, enrofloxacin significantly improved the villus height in the ileum, but the crypt depth was deeper for the duodenum in the Enro group.

**Table 5:** Effects of antimicrobial peptide WK3 on the small intestinal morphology of weaned pigs

Item	Treatment				Standard error of the mean (SEM)	*P*-value
	Blank	Control	Enro	WK3		
No. of piglets	8	8	8	8
**Duodenum**						
Villus height, μm Crypt depth, μm Villus height/crypt depth	178.52 87.70^a^ 2.12	179.39 110.85^bc^ 1.64	243.14 126.16^c^ 2.01	184.03 100.44^ab^ 1.93	10.88 4.64 0.14	0.091 0.015 0.671
**Jejunum**						
Villus height, μm Crypt depth, μm Villus height/crypt depth	217.08 100.37 2.17	173.19 112.66 1.60	201.42 97.32 1.89	196.32 83.11 2.16	6.11 4.56 0.11	0.065 0.141 0.198
**Ileum**						
Villus height, μm Crypt depth, μm Villus height/crypt depth	224.30^b^ 100.08 2.29	166.29^a^ 109.16 1.53	221.25^b^ 112.82 1.99	221.86^b^ 96.11 2.40	8.57 4.43 0.13	0.022 0.557 0.057

a,b,c Means in the same row with different superscripts differ (*P* < 0.05).

All the values are expressed as means ± SEM.

### Bacterial numbers

As shown in Table 6, the cell counts of *Enterococcus* spp. were lower in piglets from the WK3 group than in those from the control group (*P* < 0.01) but not significantly different from the blank group. In the WK3 group, *Enterobacterium* spp. showed a tendency to decrease (0.05 < *P* < 0.01), and there was no significant effect on the total number of bacteria, number of *Lactobacillus* spp. or number of *Bifidobacteria* spp. Antibiotic treatment significantly decreased the number of *Enterococcus* spp. (*P* < 0.01) in the cecal contents.

**Table 6:** Effects of WK3 on bacterial numbers in the cecal digesta of piglets

Item	Treatment				Standard error of the mean (SEM)	*P*-value
	Blank	Control	Enro	WK3		
No. of piglets	8	8	8	8		
Total eubacteria	18.26	15.05	14.65	14.33	0.240	0.510
*Enterobacterium* spp.	3.16	4.67	3.91	3.51	0.21	0.054
*Enterococcus* spp.	12.18^b^	13.68^b^	3.47^a^	3.87^a^	1.25	<0.001
*Lactobacillus* spp.	12.92	10.98	12.61	12.48	0.34	0.174
*Bifidobacteria* spp.	11.29	10.07	10.51	10.06	0.27	0.357

a,b Means in the same row with different superscripts differ (*P* < 0.05).

All the values are expressed as means ± SEM.

### Pro-inflammatory cytokine expression in the jejunal mucosa

Table 7 shows that the mRNA expression levels of *IL-1α* (*P* < 0.01), *TLR-4* (*P* < 0.05) and *MyD88* (*P* < 0.01) in the jejunal mucosa of the WK3 group were lower than those of the control group. There were differences in the mRNA expression levels of *IL-1β* (*P* > 0.05) and *IL-8* (*P* > 0.05) in the jejunal mucosa between the WK3 group and the control group. However, differences in the expression levels of *IL-1α*, *IL-1β, IL-*8, *TLR-4,* and *MyD88* in the jejunal mucosa between the WK3 and the blank group were not significant. Compared with the control group, treatment with antibiotics also significantly decreased the mRNA expression levels of *IL-8* (*P* < 0.05) and *TLR-4* (*P* < 0.01) in the jejunal mucosa, whereas the mRNA expression levels of *IL-1β* (*P* < 0.01) significantly increased.

**Table 7:** Effects of WK3 on the messenger ribonucleic acid expression of cytokines in the jejunal mucosa of piglets

Item	Treatment				Standard error of the mean (SEM)	*P*-value
	Blank	Control	Enro	WK3		
No. of piglets	8	8	8	8		
Interleukin-1α (*IL-1*α)	0.56^ab^	1.00^c^	0.71^bc^	0.38^a^	0.06	<0.001
Interleukin-1β (*IL-1*β)	1.01^a^	1.01^a^	1.41^b^	0.74^a^	0.07	0.005
Interleukin-8 (*IL-8*)	0.69^ab^	1.06^b^	0.54^a^	0.97^b^	0.08	0.045
Toll-like receptors 4 (*TLR-4*)	0.53^a^	1.15^b^	0.42^a^	0.36^a^	0.09	0.002
Myeloid differentiation primary response 88 (*MyD88*)	0.60^a^	1.17^c^	0.76^abc^	0.67^ab^	0.08	0.044

a,b,c Means in the same row with different superscripts differ (*P* < 0.05).

All the values are expressed as means ± SEM.

### Antioxidant capacity of the jejunum

The effects of WK3 on the antioxidant capacity of the jejunum of weaned pigs are shown in Table 8. Compared with the control group, treatment with both WK3 and antibiotics resulted in significant reductions in MDA concentrations (*P* < 0.05) and increased SOD activity (*P* <0.05) in the jejunum. However, no changes in T-AOC (*P* > 0.05) or GSH-Px activity (*P* > 0.05) were observed in the jejunum after treatment with WK3.

**Table 8:** Effects of antimicrobial peptide WK3 on antioxidant capacity in the jejunum of weaned pigs

Item^1^	Treatment				Standard error of the mean (SEM)	*P*-value
	Blank	Control	Enro	WK3		
No. of piglets	8	8	8	8		
Total antioxidant capacity (T-AOC), U/mgprot	0.54	0.65	0.56	0.59	0.02	0.421
Glutathione peroxidase (GSH-Px), U/mgprot	25.23	22.86	25.83	24.65	1.13	0.831
Superoxide dismutase (SOD), U/mgprot	13.79^c^	10.05^a^	11.99^b^	12.14^bc^	0.34	0.003
Malondialdehyde (MDA), nmol/mgprot	0.91^ab^	1.71^c^	0.93^b^	0.81^a^	0.15	0.036

a,b,c Means in the same row with different superscripts differ (*P* < 0.05).

All the values are expressed as means ± SEM.

## Discussion

### Diarrhea and growth performance

Diarrhea is a condition frequently reported in weaned piglets, which severely affects growth performance of piglets and restricts the development of pig farming (15). Enrofloxacin has been regarded as a therapeutic agent for treating diarrhea in weaned pigs, which is not suitable for modern agriculture due to its resistance and residue. In this study, both WK3 and enrofloxacin effectively exerted bactericidal activity *in vivo* for treating diarrhea of weaned piglets caused by *E. coli* K88. Yi et al. also reported that cathelicidin-WA showed a similar efficacy to enrofloxacin in reducing diarrhea index, which was consistent with this study (16). In addition, the results showed that WK3 and enrofloxacin enhance the growth performance of weaned piglets. Some previous studies have found that the dietary supplementation of AMPs had positive effects on growth performance (12, 17, 18). Feng et al. demonstrated that pigs injected with 0.6 mg/kg cathelicidin-BF increased ADFI and ADG of weaned piglets during the 7 days experimental period (19). The effects of AMPs on growth performance could be explained based on their antimicrobial activity and improvement of nutrient digestibility. A previous study showed that colicins E1 and N hampered the activities of *E. coli* strains, which caused postweaning diarrhea and edema disease in pigs (20). It was reported that diets supplemented with AMP-A3 or P5 in pigs improved digestibility of dry matter, crude protein and gross energy (17, 21).

### Villus height and crypt depth

Histomorphological features of the small intestine, such as villus height, crypt depth, and their ratio, are important indicators of gut health in pigs. A healthy gut is characterized by a high ratio of villus height to crypt depth (22). Toxins released by pathogenic bacteria into the intestine lead to intestinal mucosal inflammation and morphological changes associated with diarrhea. *Escherichia coli* K88 can disrupt the small intestine villi and cause diarrhea. Weaning and *E. coli* infections not only lead to atrophy of the intestinal villi but also deepen the crypts of piglets.

In the current study, the application of WK3 increased villus height in the ileum. A previous report also showed that the jejunum and duodenum of weanling pigs fed a diet supplemented with AMP-A3 showed an increased villus height and villus height to crypt depth ratio (17). Tang et al. reported an increase in villus height and villus height to crypt depth ratio in the jejunum and ileum of pigs, which were fed a diet supplemented with lactoferricin and lactoferrampin (23). In contrast, Jin et al. reported that dietary supplementation with potato AMPs did not induce changes in the intestinal morphology of weaning pigs (24). This discrepancy in results might be due to differences in the species and origin (natural or synthetic) of AMPs. Generally, intestinal morphology indicates intestinal health status (25). Greater villus height increases the surface area for the absorption of nutrients. Increased villus height and villus height to crypt depth ratio are directly correlated with greater epithelial turnover (26). In this study, greater intestinal absorption due to increased villus height might be the reason why WK3 improved growth performance of piglets. These findings suggested that AMPs could be used as growth promoters in piglet production.

### Bacterial numbers

Balanced intestinal flora are important for the growth and health of piglets; the intestinal flora may range from simple to complex and will gradually stabilize with age (27, 28). The most important beneficial effect of endogenous microflora is the creation of a barrier that makes it more difficult for exogenous pathogenic bacteria to colonize the gastrointestinal tract, which is a phenomenon known as colonization resistance (29). The intestinal microbiota also has essential functions in host metabolism and in directing immune system development (30). Because of the difference in the intestinal physiological environment and metabolism, the diversity of bacteria in the cecum increased significantly than that in the small intestine (31). Dietary supplementation with AMPs can improve the microbiota composition in the intestines of weaned pigs. Yoon et al. reported that pigs that fed an AMP-P5 supplemented diet had fewer ileal and cecal total anaerobic bacteria, including *Clostridium* spp. and *coliforms* (21). In addition, cecropin AD supplementation increased the number of beneficial *Lactobacillus* spp. in the cecum (11). In this study, the application of WK3 resulted in a significant decrease in the number of *Enterobacterium* spp. and *Enterococcus* spp. in the cecum of piglets. This finding was consistent with those of previous studies, suggesting that WK3 reduced diarrhea by decreasing the number of harmful bacteria in the cecum.

### Pro-inflammatory cytokine expression

Although various factors may cause diarrhea, intestinal inflammation remains the common symptom of this disorder. Pro-inflammatory cytokine levels, such as those of *IL-6* and *TNF-α*, typically increase in the small intestines of piglets after weaning (32). In this study, we demonstrated that WK3 suppressed intestinal inflammation by downregulating the mRNA expression of *IL-1α*, *TLR-4* and *MyD88* in the jejunal mucosa. The first function of *TLR4* is to recognize exogenous molecules from pathogens. In addition, *TLR4* activity involves the recognition of endogenous molecules released by damaged tissues and necrotic cells. *MyD88* is an important transduction protein in toll like receptors. Similar to our results, previous studies have shown that cathelicidin-BF significantly decreased the expression levels of *IL-6, IL-8* and *IL-22* (33). In addition, Song et al. reported that cathelicidin-BF pretreatment significantly reduced *TNF-α* mRNA levels compared with those in lipopolysaccharides-treated mice (34). Collectively, our results suggested that the mechanisms by which WK3 inhibited inflammation could be mediated by the downregulation of expression of inflammatory factors. This effect may explain why WK3 and enrofloxacin elicit similar inhibition of intestinal inflammation in weaned piglets with diarrhea.

### Antioxidant capacity

Oxidative stress and inflammation are highly correlated. Antioxidant enzymes are the first layer of defense (35), and the main antioxidant enzymes in the body include SOD, GSH-Px, and Catalase (CAT) (36). SOD catalyzes the transformation of superoxide radicals into H_2_O_2_ and O_2_, and is the first enzyme that interacts with oxyradicals (37). GSH serves as a first-line defense of the body against tissue injury by releasing chemicals through its ROS scavenging, cell viability-enhancing and membrane-stabilizing effects (38). MDA is an end product of free-radical chain reactions and lipid peroxidation, and is frequently used in the measurement of lipid peroxide levels. T-AOC is a comprehensive reflection of the enzymatic and nonenzymatic antioxidant capacity in the body (39). A previous study has demonstrated that AMPs increased GSH-Px content, SOD activity and the T-AOC in the serum of piglets (20). In this study, WK3 supplementation resulted in a significant reduction of MDA concentrations and an increase of T-SOD activity in the jejunum. Our results also demonstrated that WK3 could alleviate intestinal oxidative damage induced by *E. coli*.

## Conclusion

The results of this study showed that WK3 improved growth performance and reduced the incidence of diarrhea in piglets challenged with ETEC K88. These findings could be attributed to the antimicrobial activity of WK3 *in vivo* and the immune response reduction that occurred due to regulation of the secretion and expression of cytokines. Moreover, WK3 had positive effects on intestinal probiotics and oxidative damage in piglets. Therefore, it appears that WK3 has the potential to be utilized as an alternative to antibiotics administered as a dietary supplement for weaned piglets.

## References

[1] Rhouma M, Beaudry F, Theriault W, Bergeron N, Beauchamp G, Laurent-Lewandowski S, et al. *In vivo* therapeutic efficacy and pharmacokinetics of colistin sulfate in an experimental model of enterotoxigenic *Escherichia coli* infection in weaned pigs. Vet Res 2018; 47: 58. doi: 10.1186/s13567-016-0344-y PMC4884413 27234971

[2] Rhouma M, Fairbrother JM, Beaudry F, Letellier A. Post weaning diarrhea in pigs: risk factors and non-colistin-based control strategies. Acta Vet Scand 2017; 59: 31. doi: 10.1186/s13028-017-0299-7 28526080 PMC5437690

[3] Li H, Zhang L, Chen L, Zhu Q, Wang W, Qiao J. *Lactobacillus acidophilus* alleviates the inflammatory response to enterotoxigenic *Escherichia coli* K88 via inhibition of the NF-κB and p38 mitogen-activated protein kinase signaling pathways in piglets. BMC Microbiol 2016; 16: 273. doi: 10.1186/s12866-016-0862-9 27832756 PMC5105324

[4] Wang WW, Ma H, Yu HJ, Qin GY, Tan ZF, Wang YP, et al. Screening of *Lactobacillus plantarum* subsp. plantarum with potential probiotic activities for inhibiting ETEC k88 in weaned piglets. Molecules 2020; 25(19): e4481. doi: 10.3390/Molecules25194481 33003556 PMC7582832

[5] Sen S, Ingale SL, Kim YW, Kim JS, Kim KH, Lohakare JD, et al. Effect of supplementation of *Bacillus subtilis* LS 1-2 to broiler diets on growth performance, nutrient retention, caecal microbiology and small intestinal morphology. Res Vet Sci 2012; 93: 264–8. doi: 10.1016/j.rvsc.2011.05.021 21757212

[6] Manafi M, Hedayati M, Pirany N, Omede AA. Comparison of performance and feed digestibility of the non-antibiotic feed supplement (NovacidTM) and an antibiotic growth promoter in broiler chickens. Poult Sci 2019; 98(6): 698. doi: 10.3382/ps/pey529 30285253

[7] Zhang L, Li G, Zhan N, Sun T, Cheng B, Li Y, et al. Expression of a *Pseudomonas aeruginosa*-targeted antimicrobial peptide T9W in *Bacillus subtilis* using a maltose-inducible vector. Process Biochem 2019; 81: 22–7. doi: 10.1016/j.procbio.2019.03.008

[8] Da CN, Cobacho NB, Viana JF, Lima LA, Sampaio KB, Dohms SS, et al. The next generation of antimicrobial peptides (AMPs) as molecular therapeutic tools for the treatment of diseases with social and economic impacts. Drug Discov Today 2017; 22: 234–48. doi: 10.1016/j.drudis.2016.10.017. 27890668 PMC7185764

[9] Wang J, Dou X, Song J, Lyu Y, Zhu X, Xu L, et al. Antimicrobial peptides: promising alternatives in the post feeding antibiotic era. Med Res Rev 2019; 39(3): 831–59. doi: 10.1002/med.21542 30353555

[10] Xu L, Shao C, Li G, Shan A, Chou S, Wang J, et al. Conversion of broad-spectrum antimicrobial peptides into species-specific antimicrobials capable of precisely targeting pathogenic bacteria. Sci Rep 2020; 10(1): 994. doi: 10.1038/s41598-020-58014-6 31969663 PMC6976587

[11] Liu YL, Ma AJ, Han PP, Chen Z, Jia YM. Antibacterial mechanism of brevilaterin B: an amphiphilic lipopeptide targeting the membrane of Listeria monocytogenes. Appl Microbiol Biot 2020; 104: 10531–39. doi: 10.1007/s00253-020-10993-2 33170327

[12] Wu S, Zhang F, Huang Z, Liu H, Xie C, Zhang J, et al. Effects of the antimicrobial peptide cecropin AD on performance and intestinal health in weaned piglets challenged with *Escherichia coli*. Peptides 2012; 35: 225–30. doi: 10.1016/j.peptides.2012.03.030 22490448

[13] Xu L, Chou S, Wang J, Shao C, Li W, Zhu X, et al. Antimicrobial activity and membrane-active mechanism of tryptophan zipper-like β-hairpin antimicrobial peptides. Amino Acids 2015; 47: 2385–97. doi: 10.1007/s00726-015-2029-7 26088720

[14] NRC. Nutrient requirements of swine. National Academies Press. Washington, DC; 2012.

[15] Yu J, Song Y, Yu B, He J, Zheng P, Mao X, et al. Tannic acid prevents post-weaning diarrhea by improving intestinal barrier integrity and function in weaned piglets. Anim Reprod Sci 2020; 11: 87. doi: 10.1186/s40104-020-00496-5 PMC7460753 32884745

[16] Yi H, Zhang L, Gan Z, Xiong H, Yu C, Du H, et al. High therapeutic efficacy of Cathelicidin-WA against postweaning diarrhea via inhibiting inflammation and enhancing epithelial barrier in the intestine. Sci Rep 2016; 6: 25679. doi: 10.1038/srep25679 27181680 PMC4867772

[17] Yoon JH, Ingale SL, Kim JS, Kim KH, Lee SH, Park YK. Effects of dietary supplementation of antimicrobial peptide-A3 on growth performance, nutrient digestibility, intestinal and fecal microflora and intestinal morphology in weanling pigs. Anim Feed Sci Technol 2012; 177: 98–107. doi: 10.1016/j.anifeedsci.2012.06.009

[18] Xiong X, Yang H, Li L, Wang Y, Huang R, Li F, et al. Effects of antimicrobial peptides in nursery diets on growth performance of pigs reared on five different farms. Livest Sci 2014; 167: 206–10. doi: 10.1016/j.livsci.2014.04.024

[19] Feng J, Wang L, Xie Y, Chen Y, Yi H, He D. Effects of antimicrobial peptide cathelicidin-BF on diarrhea controlling, immune responses, intestinal inflammation and intestinal barrier function in piglets with postweaning diarrhea. Int Immunopharmacol 2020; 85: 106658. doi: 10.1016/j.intimp.2020.106658 32531710

[20] Wang JH, Wu CC, Feng J. Effect of dietary antibacterial peptide and zinc-methionine on performance and serum biochemical parameters in piglets. Czech J Anim Sci 2011; 56: 30–6. doi: 10.17221/341/2009-CJAS

[21] Yoon JH, Ingale SL, Kim JS, Kim KH, Lohakare J, Park YK, et al. Effects of dietary supplementation with antimicrobial peptide-P5 on growth performance, apparent total tract digestibility, faecal and intestinal microflora and intestinal morphology of weanling pigs. J Sci Food Agric 2013; 93: 587–92. doi: 10.1002/jsfa.5840 22903784

[22] Niu Y, He J, Zhao Y, Gan Z, Shen M, Zhang L, et al. Dietary enzymatically treated Artemisia annua L. supplementation improved growth performance and intestinal antioxidant capacity of weaned piglets. Livest Sci 2020; 232: 103937. doi: 10.1016/j.livsci.2020.103937

[23] Tang Z, Yin Y, Zhang Y, Huang R, Sun Z, Li T, et al. Effects of dietary supplementation with an expressed fusion peptide bovine lactoferricin-lactoferrampin on performance, immune function and intestinal mucosal morphology in piglets weaned at age 21 d. Br J Nutr 2009; 101: 998–1005. doi: 10.1017/S0007114508055633 18840311

[24] Jin Z, Yang YX, Choi JY, Shinde PL, Yoon SY, Hahn TW, et al. Effects of potato (*Solanum tuberosum* L. cv. Golden valley) protein having antimicrobial activity on the growth performance, and intestinal microflora and morphology in weanling pigs. Anim Feed Sci Technol 2008; 140: 139–54. doi: 10.1016/j.anifeedsci.2007.12.006

[25] Caspary WF. Physiology and pathophysiology of intestinal absorption. Am J Clin Nutr 1992; 55: 299S–308S. doi: 10.1093/ajcn/55.1.299s 1728844

[26] Fan YK, Croom J, Christensen VL, Black BL, Bird AR, Daniel LR, et al. Jejunal glucose uptake and oxygen consumption in turkey poults selected for rapid growth. Poult Sci 1997; 76: 1738. doi: 10.1093/ps/76.12.1738 9438290

[27] Wang R, Yu Hao, Fang H, Jin Y, Zhao Y, Shen J, et al. Effects of dietary grape pomace on the intestinal microbiota and growth performance of weaned piglets. Arch Anim Nutr 2020: 1–13. doi: 10.1080/1745039X.2020.1743607 32308036

[28] Mathew AG, Upchurch WG, Chattin SE. Incidence of antibiotic resistance in fecal *Escherichia coli* isolated from commercial swine farms. J Anim Sci 1998; 76: 429–34. doi: 10.2527/1998.762429x 9498348

[29] Brassart D, Schiffrin EJ. The use of probiotics to reinforce mucosal defence mechanisms. Trends Food Sci Technol 1997; 8: 321–6. doi: 10.1016/S0924-2244(97)01071-6

[30] Brown K, Decoffe D, Molcan E, Gibson DL. Diet-induced dysbiosis of the intestinal microbiota and the effects on immunity and disease. Nutrients 2012; 4: 1552–3. doi: 10.3390/nu4081095 PMC3448089 23016134

[31] Suchodolski JS, Camacho J, Steiner JM. Analysis of bacterial diversity in the canine duodenum, jejunum, ileum, and colon by comparative 16S r RNA gene analysis. FEMS Microbiol Ecol 2008; 66(3): 567–78. doi: 10.1111/j.1574-6941.2008.00521.x 18557939

[32] Wang J, Tian S, Yu H, Wang J, Zhu W. Response of colonic mucosa-associated microbiota composition, mucosal immune homeostasis, and barrier function to early life galactooligosaccharides intervention in suckling piglets. J Agric Food Chem 2019; 67: 578–88. doi: 10.1021/acs.jafc.8b05679 30562014

[33] Yi H, Yu C, Zhang H, Song D, Jiang D, Du H, et al. Cathelicidin-BF suppresses intestinal inflammation by inhibiting the nuclear factor-kappaB signaling pathway and enhancing the phagocytosis of immune cells via STAT-1 in weanling piglets. Int Immunopharmacol 2015; 28: 61–9. doi: 10.1016/j.intimp.2015.05.034 26044347

[34] Song D, Zong X, Zhang H, Wang T, Yi H, Luan C, et al. Antimicrobial peptide Cathelicidin-BF prevents intestinal barrier dysfunction in a mouse model of endotoxemia. Int Immunopharmacol 2015; 25: 141–7. doi: 10.1016/j.intimp.2015.01.017 25639228

[35] Wang J, Tian S, Wang J, Zhu W. Early galactooligosaccharide intervention alters the metabolic profile, improves the antioxidant capacity of mitochondria and activates the AMPK/Nrf2 signaling pathway in suckling piglet liver. Food Funct 2020; 11: 7280–92. doi: 10.1039/d0fo01486a 32779675

[36] Doyotte A, Cossu C, Jacquin MC, Babut M, Vasseur P. Antioxidant enzymes, glutathione and lipid peroxidation as relevant biomarkers of experimental or field exposure in the gills and the digestive gland of the freshwater bivalve Unio tumidus. Aquat Toxicol 1997; 39: 93–110. doi: 10.1016/S0166-445X(97)00024-6

[37] Cheung CC, Zheng GJ, Li AM, Richardson BJ, Lam PK. Relationships between tissue concentrations of polycyclic aromatic hydrocarbons and antioxidative responses of marine mussels, Perna viridis. Aquat Toxicol 2001; 52: 189. doi: 10.1016/S0166-445X(00)00145-4 11239681

[38] Wei B, Nie S, Meng Q, Qu Z, Shan A, Chen Z. Effects of L-carnitine and/or maize distillers dried grains with solubles in diets of gestating and lactating sows on the intestinal barrier functions of their offspring. Br J Nutr 2016; 116: 459–69. doi: 10.1017/S0007114516001951 27256481

[39] Liu Y, Han J, Huang J, Wang X, Wang F, Wang J. Dietary L-arginine supplementation improves intestinal function in weaned pigs after an *Escherichia coli* lipopolysaccharide challenge. Asian Australas J Anim 2009; 22: 1667–75. doi: 10.5713/ajas.2009.90100

